# ‘What Do People With Long Covid Want From Healthcare Services?’ A Qualitative Exploration From Lived Experience

**DOI:** 10.1111/hex.70607

**Published:** 2026-02-24

**Authors:** Clare Rayner, Nikki Smith, Ruairidh Milne, Ghazala Mir, Johannes de Kock, Nawar Diar Bakerly, The LOCOMOTION Consortium, Nawar Bakerly, Kumaran Balasundaram, Megan Ball, Mauricio Barahona, Alexander Casson, Jonathan Clarke, Karen Cook, Rowena Cooper, Vasa Curcin, Julie Darbyshire, Helen Davies, Helen Dawes, Simon de Lusignan, Brendan Delaney, Carlos Echevarria, Sarah Elkin, Ana Belen Espinosa Gonzalez, Rachael Evans, Sophie Evans, Zacchaeus Falope, Ben Glampson, Madeline Goodwin, Trish Greenhalgh, Darren C. Greenwood, Stephen Halpin, Juliet Harris, Will Hinton, Mike Horton, Samantha Jones, Joseph Kwon, Cassie Lee, Ashliegh Lovett, Mae Mansoubi, Victoria Masey, Harsha Master, Erik Mayer, Bernardo Meza‐Torres, Ruairidh Milne, Ghazala Mir, Jacqui Morris, Adam Mosley, Jordan Mullard, Daryl O'Connor, Rory O'Connor, Thomas Osborne, Amy Parkin, Stavros Petrou, Anton Pick, Denys Prociuk, Clare Rayner, Amy Rebane, Natalie Rogers, Janet Scott, Manoj Sivan, Nikki Smith, Adam Smith, Emma Tucker, Ian Tucker‐Bell, Paul Williams, Darren Winch, Conor Wood

**Affiliations:** ^1^ Patient Advisory Group Co‐Lead, Locomotion Project Manchester UK; ^2^ Patient Advisory Group Member, Locomotion Project Windsor UK; ^3^ Patient Advisory Group Co‐Lead, Locomotion Project University of Southampton Southampton UK; ^4^ Leeds Institute of Health Sciences University of Leeds Leeds UK; ^5^ NHS Highlands, COVID Recovery Services Inverness UK; ^6^ School for Psychosocial Research North West University Potchefstroom South Africa; ^7^ Northern Care Alliance Manchester UK; ^8^ Manchester Metropolitan University Manchester UK

**Keywords:** COVID clinic, COVID‐19, long COVID, post COVID syndrome, SARS‐CoV2

## Abstract

**Background:**

Long COVID (LC) is a chronic, multisystem condition affecting millions globally, with significant personal, social and economic consequences. Despite increasing recognition of its impact, healthcare services for LC remain inconsistent with patients frequently encountering fragmented services, scepticism and delays leading to patient‐voiced frustration. Therefore, understanding patient priorities is crucial for optimising service provision.

**Objectives:**

To explore what individuals with LC want from healthcare services—drawing on their lived experience and collaborative insights with clinicians and researchers, to inform principles for improving care delivery, barriers to access, expectations for service improvement, and the role of multidisciplinary care in managing LC.

**Methods:**

A qualitative study using thematic analysis was conducted, incorporating multiple data sources, including semi‐structured interviews, workshops, and a patient‐led audit. Key themes were identified, focusing on healthcare access, clinical assessments, treatment options, and service organisation.

**Study Participants:**

Twenty‐seven LC sufferers from the LOCOMOTION Patient Advisory Group (PAG) and Patient Advisory Network (PAN), along with clinicians and researchers involved in LC service provision across the United Kingdom, participated in the study.

**Results:**

Three major themes emerged: (1) Who the services are for: Equity of access for all those with LC. Barriers such as stigma, inequitable access and lack of clinician awareness need to be addressed. (2) What services should do: Consistent and standardised assessments and diagnostic clarity—particularly for modifiable conditions like autonomic dysfunction—and an emphasis on the need for early medical intervention, not just rehabilitation. (3) How services should operate: Care should be coordinated, proactive and adaptable to evolving evidence. Patients should not be discharged without ongoing review. Multidisciplinary collaboration should be patient‐centred and informed by up‐to‐date research.

**Conclusions:**

LC services should be designed to provide equitable, standardised and evidence‐based care. Early intervention, appropriate medical testing and sustained follow‐up are critical to improving patient outcomes. Patients emphasised the importance of being heard and the value of receiving timely care that reflects the latest scientific understanding and recognises their condition as real, treatable and deserving of ongoing clinical attention. Incorporating these insights into healthcare design may improve outcomes, service efficiency and trust between patients and providers.

**Patient and Public Contribution:**

Patients led all phases of this study, including design, analysis and writing, through active co‐production with the LOCOMOTION research team. The paper was born out of discussions within the LOCOMOTION study's Patient Advisory Group (PAG). It was taken forward by C.R., N.S. and R.M., all members of the PAG, working closely with N.B., H.d.K. and G.M.

## 1 Introduction

Long COVID (LC) or post‐COVID condition (PCC) in adults is defined by the World Health Organisation (WHO) as occurring ‘in individuals with a history of probable or confirmed SARS‐CoV‐2 infection usually 3 months from the onset of COVID‐19 with symptoms that last for at least 2 months and cannot be explained by an alternative diagnosis’ [1]. Symptoms may be new onset, following initial recovery, or persist from the initial acute illness. Symptoms may also fluctuate or relapse over time. Common symptoms include fatigue, shortness of breath, cognitive dysfunction but also others which have an impact on everyday functioning [2]. In essence, LC is a chronic, multi‐faceted condition following SARS‐CoV‐2 infection, often without an alternative explanation for the persistent symptoms [3]. Such wide‐ranging effects underscore that LC is a syndrome capable of impacting numerous bodily systems simultaneously [4]. LC has emerged as a significant global public health issue with an estimate of 65 million people affected worldwide and a prevalence of 3%–6% in the United Kingdom (UK) [5, 6].

Beyond health, the societal and economic impacts of LC are profound. Many LC sufferers are unable to work or require reduced working hours. In the United Kingdom, LC costs government and wider society billions of pounds each year, including through productivity losses and greatly increased healthcare utilisation [7, 8].

There are many gaps in healthcare and the support provided for LC patients [9]. Studies have advocated for a multidisciplinary approach to the assessment and management of LC, and for this to be both contextually appropriate and tailored to the specific complexities of this multisystem condition, [10] yet current practice still lacks evidence‐based treatments [11]. In the United Kingdom, healthcare services for LC have been inequitable in terms of funding and access, and long term funding for these services remains uncertain, [12] with some clinics having closed and others having cut back on what they offer. The situation appears to be similar in other countries with disparate, non‐standardised services for LC [13]. Access to diagnosis and support has been identified as particularly difficult for those within disadvantaged groups [14]. To date, research on LC has focused on epidemiology, pathophysiology, rehabilitation, and clinical characteristics, with far fewer studies delving into patients’ lived experiences, needs and challenges. Perspectives on LC services have been largely limited to post‐hospital patients [15]. Patients and advocates have called for more attention to the personal and social dimensions of LC to be an essential ingredient for advancing research and care [4].

This study therefore aimed to address these gaps by focusing on the experiences and priorities of people with LC. By elevating the voices of those affected, our goal is to inform more responsive healthcare services and policies—ensuring that support for LC aligns with what patients themselves identify as most important.

## 2 Methods

### 2.1 Study Design

This study is a qualitative study from the LC multidisciplinary consortium optimising treatments and services across the NHS (LOCOMOTION) study in the United Kingdom. LOCOMOTION was funded by the NIHR and aimed to ‘identify the best way to treat and support the 1.9 million people in the United Kingdom now living with Long COVID’ [16]. This study involved 10 LC clinics from across the United Kingdom. Full protocol has been previously published [17].

### 2.2 Reporting Standards

This study follows the Consolidated Criteria for Reporting Qualitative Research (COREQ) checklist to ensure transparent and complete reporting of the qualitative components [18]. The framework informed both study design and the reporting of interviews, focus groups, researcher reflexivity, and data analysis.

### 2.3 Study Participants

Participants were from the LOCOMOTION Patient Advisory Group (PAG), Patient Advisory Network (PAN) and External Advisory Group (EAG) [17]. The PAG had 10 members from a variety of cultural, ethnic and socio‐economic groups. The PAG helped design LOCOMOTION, ensuring it was aligned with patients' key priorities and had a governance role. PAG members, who had the status of co‐researchers, brought lived experience, information and experience from running patient support groups, advocacy, and LC experience. Some had been patient representatives on the NHS England LC Taskforce and led national audits on access to LC clinics. The PAN comprised a total of 20 people, up to two Patient and Public Involvement (PPI) members from each participating NHS site, ensuring the demographics of each site were represented. The PAN and EAG formed part of the governance structure for LOCOMOTION and had two main purposes: to gather insights from patients about their experiences of accessing a LC clinic; and to gather insight about the experiences of people living with LC in their local communities who had not been able to access LC clinics. PAG members also took feedback on findings from the inequalities leads.

### 2.4 Participant Sampling

We used a purposive sampling strategy to ensure diversity in terms of gender, ethnicity, education, and lived experience. All participants were drawn from the LOCOMOTION consortium, including PAG, PAN, EAG, and attendees of two workshops and a patient‐led audit meeting. All members of PAG, PAN, and EAG who participated in meetings or workshops were included in the analysis. The approach was inclusive encompassing all available data across the four sources: semi‐structured interviews, two workshops, and the PAG‐led clinical audit presented at the Quality QIC meeting. Using multiple data sources added methodological richness and triangulation.

### 2.5 Research Team and Data Collection

The research team was multidisciplinary and included LC advocates (C.R., N.S., R.M.), an academic with expertise in health inequalities (G.M.) and healthcare professionals (H.d.K., N.B.).

Data were collected using four sources with the aim of purposively sampling a representative group of participants from the PAG, PAN and broader LOCOMOTION consortium:

1. Semi‐structured interviews with all patients from the PAN attending two meetings in February 2022 at 6 months into the project and two meetings in February 2023 at 18 months into the project. The PAN meetings were via online video platforms and facilitated by the two PAG Co‐Leads (CR, RM) and the PPI Support Manager. Interviews were recorded on Zoom and minutes were noted by the PPI Support Manager (PAN participants are described in Table 1).
2. Semi‐structured interviews with PAG members in June 2023 and led by PAG Co‐Lead (CR) with 6 PAG members who responded to the invitation. Interviews were conducted via online video platform, followed by email communication to ensure comments were captured accurately. A PAG member created a video of PAG members speaking their answers.
3. A workshop for staff, researchers and patients (10 participants) in July 2023 with hybrid attendance format. The workshop was facilitated by CR and the PPI Manager, with note‐taking by a second PPI Manager. Participants of the workshop are shown in Table 1. Three questions were discussed:

− What has worked in clinics that were part of LOCOMOTION?
− What has not worked in clinics that were part of LOCOMOTION?
− What elements would we keep in an ideal world?

4. A Quality Improvement Collaborative (QIC) Workshop in November 2022 held to discuss the recommendations from a PAG‐led audit of the clinics involved in LOCOMOTION [19]. The audit covered the structures and processes in LOCOMOTION LC clinics. It was designed and analysed by PAG Co‐Lead C.R., and results checked by all PAG members then written up by PAG member N.S. It followed a clinical audit format and was completed in September 2022. The clinical standards used were the WHO Living guideline on rehabilitation of Post Covid‐19 condition, World Physiotherapy Statement no.9 and NIICE guidance NG188 [9, 20, 21]. The results and recommendations of the audit were presented by PAG members to the Chief Investigators for LOCOMOTION then discussed with researchers and clinicians at the patient‐led QIC meeting in November 2022. Participants' characteristics of the PAG, PAN and QIC workshops are shown in Table 1.

**Table 1:** Demographics of the study participants.

Patient Advisory Group (PAG) (10 people) and External Advisory Group (EAG) (2 people) characteristics	Participants *N* = 12
Female/male	6/6
Protected characteristics (race, disability, sexuality)	4
Clinically (extremely) vulnerable to the effect of COVID (CEV)	1
Hospitalised with acute COVID	1
1st or higher degree or equivalent	8
**Patient Advisory Network (PAN) meetings**	
Female/male	11/4
No. with protected characteristics (race, disability, sexuality)	4
Hospitalised with acute COVID	0
**QIC workshop November 2022**	**20 participants**
Patients (PAG members)	6
Patient support manager	1
Research team members (non‐clinical)	6
Principal investigators (clinical)	2
Clinical research fellows	5
**Workshop July 2023**	**10 participants**
Patients (PAG members)	3
Patient support managers (acted as note‐taker)	2
Senior clinicians (consultants)	3
Data analysts	2

### 2.6 Data Analysis

Thematic analysis was conducted using the six‐phase approach of Braun and Clarke (2006) [22] to explore factors perceived to contribute or detract from subjective quality of care:

1. Familiarisation: C.R. and R.M. reviewed all data across sources (Zoom recordings, notes, Slack messages, audit outputs).
2. Generating initial codes: C.R. developed a coding framework inductively. Codes reflected semantic and latent content.
3. Searching for themes: Initial themes were proposed collaboratively by C.R. and R.M.
4. Reviewing themes: Themes were revised and checked by N.B. for internal coherence and clarity.
5. Defining and naming themes: Themes were refined based on input from the wider PAG group and adjusted for clarity and interpretive depth.
6. Producing the report: Final theme structure aligned to ‘Who the services are for’, ‘What services should do’ and ‘How services should operate’, with subthemes nested within each.

No software was used for analysis as this was a patient‐led project.

### 2.7 Reflexivity and Intercoder Agreement

C.R., N.S. and R.M. brought lived experience to the analysis, contributing reflexive insights into the data interpretation. They met regularly to challenge assumptions and clarify interpretations. Data were analysed using Braun and Clarke's six‐phase framework for thematic analysis [22]. Initial coding and theme development were led by CR who grouped codes under nine preliminary headings. These were refined into three overarching themes by R.M., as described in the Section 10. C.R., N.S. and R.M. reviewed and agreed on these refinements via email correspondence. A final check by the last author N.B. suggested minor adjustments to theme allocation, which were accepted by all team members. Disagreements were resolved through discussion, with N.B. providing final arbitration to ensure interpretive rigour to the data.

One example of reflexive practice was the review of subthemes post‐coding: theme names were revised to better reflect participant language and experience, rather than relying on pre‐existing service frameworks. Additionally, all members of the PAG had access to reviewed interpretations and contributed to validating the coherence of findings.

## 3 Results

Results are presented in three main domains identified from the thematic analysis. (1) Who the services are for, (2) what services do and (3) how clinics operate. The full list of participant quotes is shown in the online Appendix (1).

### 3.1 Who the Services Are For—Access to Healthcare

#### 3.1.1 People With LC, However Long They Have Had It—Not Just Those Newly Identified

By the end of 2022, halfway through LOCOMOTION, it had become clear that a growing proportion of people with LC in the United Kingdom had had persistent symptoms for nearly 3 years, and research was identifying autoimmune and other associations at later stages after infection [23]. These themes were highlighted by the PAG audit, workshop and reflected patients' experiences in the LOCOMOTION wider groups.

‘It is becoming clear that a sizeable proportion of people with LC have a chronic condition that needs long term management, and the needs of these patients are different from, say, those who get better within 9 months. We need a working definition for ‘Long LC’. In terms of pathology, a starter would be anyone still unwell more than 12 months after infection. The duration becomes essential when considering the economic burden of long‐term work absence in the prime working age groups’. [PAG member]

#### 3.1.2 A Focus on Reducing Inequalities and Stigma

LOCOMOTION identified a variety of reasons that made it difficult for patients to access LC services. Evidence from primary care records, analysed as part of LOCOMOTION activities indicated that there was under referral of Asian, mixed heritage and some deprived populations to LC clinics despite higher rates of diagnosis within these groups [24]. Qualitative studies have highlighted barriers to diagnosis and referral for a range of disadvantaged populations that prevented access to adequate information and support [14].

Patients in the PAG and PAN expressed a need for a straightforward process to access clinics. It was clear that care following acute COVID‐19 infection is disjointed and requires significant effort from patients to navigate the healthcare system:

- ‘The referral process is complicated; it gets lost or rejected. You can only get through if you ring up each day or really make a nuisance of yourself’. [PAN member]
- ‘Patients often have to coordinate their own care’. [PAN member]
- ‘…some doctors not knowing that the clinics exist or not knowing how to refer a patient to them’. [PAN member]
- ‘It was very hard beginning to get doctors to understand what we were going through. When I did get results of a scan showing blood clot and heart information, I got a lot more support because the problems were visible’. [PAG member]

Several PAN patients reported that the label of ‘Long Covid’ initially prevented them from accessing a LC clinic:

- ‘I had a red mark against my name at the GP's, with written instruction “You are not allowed to contact us about this issue again”’. [PAN member]
- ‘LC is not an easy name. LC as a term closes people's minds. Some doctors said don't mention the word LC‐it's just anxiety’. [PAN member]

Clinicians at year 2 workshop identified the most serious barrier to the provision of healthcare as being changes to government funding in England from April 2024. This fear has been realised as some clinics have closed and funding diverted elsewhere. They also noted the lack of testing for acute COVID‐19 infection obscured the true prevalence of COVID‐19, and hindered monitoring clinical consequences.

### 3.2 What Services Do

This is considered under the topics of (a) systems, (b) tests and (c) treatments.

a. Systems
  The year 2 workshop focussed on systems. Staff and patients agreed the need for:
  1. A ‘bundle’ of assessment tests while waiting to attend their first LC clinic appointment, as well as advice on pacing
  2. ‘Treating Treatable Traits’, e.g., autonomic dysfunction (AD). The earlier that symptoms can be treated the better [25].
  3. A problem‐focussed approach as this ensures patients’ priorities are covered.
  4. Effective physician‐led care by those who specialise in treating multi‐system conditions.

b. Tests
  All four data sources focussed on necessary investigations based on current guidelines and research. The patient groups emphasised ‘consistency in testing and diagnosis. Currently there are a variety of healthcare professionals involved… no real consistency, it's more based upon an individual clinician's knowledge’.
  Patients described clinic content that varied widely across the United Kingdom. This geographical inconsistency is reflected in LOCOMOTION's evaluation of its QIC network which highlighted how far the services provided within a clinic depend on the type and beliefs of the lead clinician [19].

PAG members advised

- ‘above all, people need the right tests at the right time and to consider the known range of COVID‐19‐related problems’.
- ‘Other illnesses should be ruled out’.
- Patients also want ‘mental health support because of the effect on finances and work and the difficulties of living with a long‐term disease’ and ‘signposting to relevant support, e.g., via social prescribing systems’.
  PAN members also highlighted the importance of directing to other services, e.g., financial advice services, with Occupational Therapy services being especially helpful.
  In the PAG audit and PAG‐led QIC workshop, patients, clinicians and researchers aligned on the need for:
  i. assessment and monitoring of red flags.
  ii. checking for known health problems caused by SARS‐CoV‐2.
  iii. specialist referrals, commonly but not exclusively, respiratory, cardiac and neurological.
  iv. rehabilitation therapies for specific impairments.
  v. education on assessment and management of cognitive problems.

There was a range of attitudes towards the number of tests that patients would like:

- ‘The acceptability of tests is fairly high, as people are desperate’ [EAG member]
- ‘It is critical that we understand the pathology and find treatments. Meantime, we want treatable conditions to be identified and treated. We would like clinicians to keep up with the research and put this into clinical practice. For people who are not better to be reassessed at intervals and keep an open mind’. [PAG member]
- Others wanted only those tests that are ‘likely to have a yield’ [PAN member] and ‘just ones which may be useful’ [PAN member].

c. Treatments
  The PAG advocated on behalf of other patients for treatment:
  - ‘The most important thing is that research is translated into clinical practice as soon as possible’.
  - ‘We ask please hear us, involve us, but also be brave, try things that may not necessarily fit onto an NHS tick list’.
    The second PAN meeting focussed on the assessment and treatment of Autonomic Dysfunction (AD) in preparation for the PAG audit and PAG‐led QIC workshop. These represent a truly patient‐led project. Patients, clinicians and researchers agreed at the workshop that AD was the critical quality improvement gap. The outcome was to ‘focus on recommendations that would be easy for clinics to implement and have a quick impact on patients: an easy win’. [Co‐Chief Investigator]
    Comments made by PAN members about the importance of diagnosis and treatment of AD are listed in the online Appendix (1).

Patients and staff wanted to learn more about how to manage cognitive dysfunction:

- ‘… having lived with it for 2.5 years I see a difference between neurological and cognitive issues, and brain fog’. [PAG member]
- ‘My cognitive problems were not dealt with—I was told I did not have dementia’. [PAG member]
- ‘When I said that my memory was badly affected the neurologist responded by saying “don't worry about that” with a flick of his hand as if to brush it away’. [PAG member]
- ‘Speech therapy for Covid‐related speech problems needs a neuro‐cognitive approach’. [PAG member]

### 3.3 How Services Operate

#### 3.3.1 Coordination of Care

In the end of year 2 workshop analysis (2023) of what had worked well in clinics, both patients and clinicians said it was helpful when all relevant services to support LC (assessment, specialists and rehabilitation) are based within the same organisation:

- ‘We would like to provide more LC support in‐house, as it saves referral time’. [Consultant Physician]
- ‘a one‐stop shop—having everything in the same place, it's simple to touch base with all the treatment they are receiving’. [PAN member]

Because of the multiple, heterogeneous health problems caused by COVID, there are many potential referrals to specialists, tests and rehabilitation therapists. Some patients said that referral and discharge pathways lacked clarity.

- ‘I want to be informed where I am on the referral pathway, so I don't have to chase to find out—bearing in mind our cognitive dysfunction!’ [PAG member]
- ‘Medical silos are a problem—being left high and dry if a specialist discharges, instead of back to the LC service which has a good understanding of all our problems’. [PAG member]
- ‘When I was discharged from the Long COVID clinic I felt abandoned and adrift. This illness had caused me to lose my job. It was affecting my life in so many different ways’. [PAG member]
- ‘Clinics need a mechanism for getting in touch with LC patients when new treatments and tests become available. Or regular review, e.g. every year or 2 years—to look for new problems or problems that might have been missed previously’. [PAN member]

Staff suggested a system of ‘patient‐initiated follow‐up’ with the LC clinic in this situation. Or a coordinating clinic (such as in Paediatric services) who have a ‘holding pattern’, i.e., before discharge a patient could touch base with the LC service. PAG welcomed this suggestion which was taken back to some of their own clinics and worked well.

#### 3.3.2 Proactive

Patients wanted clinicians to keep up with research.

- ‘Patients shouldn't have to push for what there is already evidence of, e.g., the right tests’. [PAN member]
- ‘Most clinics are about rehab, not investigation or treatment—frustration with that. (Though specific aspects of rehab can be very helpful)’. [PAN member]

Practical suggestion included:

- ‘… advice that everyone can be given whilst waiting to be seen—early pacing advice is one of the most helpful things’. [PAN member]
- ‘It would be good to be given a sheet about POTS (postural orthostatic tachycardia syndrome) symptoms whilst you're waiting for tests. It's a good opportunity to monitor your own symptoms and try things that may help’. [PAN member]
- Patients wanted prevention of worsening health problems:
- ‘Reinfections are damaging and need to be prevented to reduce the overall impact of the illness on daily functions’. [PAG member]
- ‘…… reinfections—how to avoid (masks, ventilation, work adjustments etc.), how to manage if they happen ….’ [EAG member]
- ‘…. prevention of cardiovascular complications that are associated with LC’. [PAG member]

Digital technology such as the COVID‐19 Yorkshire Rehabilitation Scale ‘app’ (C19‐YRS) could be used more widely. Patients found it useful to visualise charts and changes and ‘it would be helpful for GPs to access’. [PAN member] Clinicians and data specialists at the clinic review workshop said, ‘newer clinics benefit from sharing best practice and existing resources so they don't have to design them from scratch themselves—collaboration is key’.

#### 3.3.3 ‘Evidence‐Based’, to Improve Patient Care, Not as a Strait Jacket

One PAG member pointed‐out ‘It is a recurring theme in patient groups that healthcare for LC lags behind the research evidence’. Another suggested ‘Education is needed so that staff are up to date on research: education influences everything—assessments, investigations/tests, advice, treatments, monitoring’.

‘We want clinicians to base their diagnostic and therapeutic decisions on best available evidence, and there is now lots…Not to be paralysed by out of date evidence such as the NICE guidelines (NICE, 2021) or the lack of Randomised Control Trials. We need clinicians to be agile and fleet‐of‐foot’. [PAG member]

‘As clinics close, there is real concern that the expertise gained over the last few years is going to be lost’. [PAG member]

#### 3.3.4 Genuinely Patient Centred, Even Multi‐Disciplinary Team Meetings (MDTs)

Patients valued the holistic approach of MDTs:

- ‘My clinic was a really good experience with a holistic approach with tests, referrals, health education and teaching of self‐management techniques’. [PAN member]
- ‘There is an obvious physical impact but there is the emotional impact as well’. [PAN member]
- ‘Our experience is much better when the patient is put at the centre of diagnosis and support, and has things properly explained to them’. [PAN member]
- ‘I think you really need to listen to your patient if they say they are completely different from before they got ill…. It should not be passed over as anxiety or needing exercise’. [PAG member]

The need to rapidly build understanding of a new condition was echoed by senior clinicians who stated that MDTs had allowed ‘Learning from colleagues, upskilling within teams, improved continuity of care and a better awareness of the full range of symptoms which apply to LC patients’. [Consultant Physician]

Patients articulated a proviso about MDTs that they can sometimes be disempowering with decisions made without patient involvement. ‘This works directly against the principle of (Nothing About Me Without Me)’ [PAG member]. In addition, ‘the term multi‐disciplinary team in my experience is often used by managers and policy makers to refer to non‐doctors. We therefore need to be careful about the term and how we use it’. [PAG member]

## 4 Discussion

Our study explored care for LC, by investigating what service users, providers and researchers think LC care should look like: who clinics should be for and what care should be provided. Although the purpose of qualitative research is not to offer a generalisable oversight of experiences, we found that, for the people we spoke to, what mattered was that LC services should be easily accessible and that they should offer joined up care, with prompt tests at the right time, as well as early advice, treatment of treatable symptoms and review of longer‐term service users. Key underpinning principles were that services should be consistent across different locations and should take account of the latest research into investigations, treatments and rehabilitation.

The patient participants in this study constituted diverse sources of evidence, and included patients with a wide range of experiences in various geographical locations. However, we acknowledge that inclusion of a larger range of both patients and clinicians from an even broader spectrum of communities, locations and socioeconomic status, may have highlighted additional individual challenges experienced in relation to LC healthcare.

## 5 Strengths and Limitations

This study provides a comprehensive account of what patients want from LC services, drawn from workshops with patients, staff and researchers. The duration of the project (3 years) captures the changing landscape of knowledge of LC from mystery to multimorbidity. Patient involvement was central to LOCOMOTION: the study protocol was designed with patients, the PAG was integral to the governance of the study and patients were closely involved in its delivery. This model led to a continual focus on patient outcomes, and we recommend this model of patient involvement for future health services research.

Strengths of this study included the patient‐led audit of LC services and recommendations on the content and delivery of clinics, despite the fragmented LC clinic system. The study highlighted the issue of ‘Long‐Long COVID’ not discussed elsewhere, and prevention of complications, such as reinfection with the SARS‐CoV‐2 virus.

Two important limitations need highlighting. First, LOCOMOTION was conceived as a study of LC clinics in 2021, but by 2024/25 the UK funding environment for LC clinics had greatly changed. Many clinics were closed, with others severely reduced or dispersed, with care taking place in a wider variety of clinical settings. However, our findings are independent of this change as they set out what LC patients want for LC care in any setting, and what should be done and how for all people with post‐COVID conditions. Second, the 27 patients involved in the study will not be fully representative of all people with LC from across the United Kingdom (for instance, in terms of language, geography, socioeconomic status, and ability to access and receive healthcare for LC). They comprised of patients from the PAG, PAN and EAG, whose roles include providing patient involvement (to varying degrees) on the overarching study, as opposed to individuals with no link to the study and no experience of research participation or involvement, who may bring independent additional perspectives. The authors did, however, obtain feedback from the inequalities work package. Furthermore, part of the overall role of the EAG included gathering insights from people with LC in local communities.

Our study sought to synthesise a wide perspective based on patients and staff opinions and experience and was based on international clinical guidelines for basic healthcare provision (assessment, investigation, red flags and treatment) for people with LC [1, 20]. The variety and degree of health problems experienced by the patient participants in this study are typical of larger patient populations. Previous research has shown that inequity of access is associated with greater healthcare needs, so the needs of participants may be less than those that research aims to reach [14, 26, 27].

## 6 UK and International Literature and Context

This work builds on previous studies indicating the importance for LC service users of easy access to services [26, 27, 28]. Not only patients, but also clinic staff and researchers, placed great value on easily accessible joined up care in LC clinics. Our research also echoes previous findings calling for an improved coordination and integration of LC services, [27] with a lack of continuity and integration of care seen as a barrier to access and detrimental to patients’ journeys through the healthcare system.

Previous research on LC services has largely focused on describing patient experiences and barriers to accessing care, often calling for improved collaboration between patients and professionals, with greater incorporation of lived experience into service design [4, 14, 15, 26, 27, 29]. These studies have provided valuable insights but have typically positioned patients as informants rather than active partners. Our study advances the field by embedding patients at every stage of the LOCOMOTION programme—from the NIHR funding application and design of work packages to governance, analysis and dissemination. This co‐production model goes beyond consultation to deliver patient‐led research initiatives, including a national audit of LC clinics against WHO rehabilitation standards and NICE guidance [1, 21]. Unlike prior work, we also highlight the need to address emerging biomedical aspects of LC, such as autonomic dysfunction and cognitive impairment, and propose practical steps for integrating these into clinical pathways. By combining lived experience with clinical and research expertise, our study offers a transferable framework for equitable, evidence‐based LC care that can inform both national policy and international service models.

## 7 Implications for Practice and Policy

Our findings highlight the need for LC services to adopt a proactive, multidisciplinary model that addresses diagnostic and treatment gaps rather than relying solely on rehabilitation. Based on patient priorities and emerging evidence, clinicians and service providers can consider:

### 7.1 Early Identification and Standardised Assessment

Implement a core bundle of investigations at first contact, including screening for autonomic dysfunction (e.g., postural orthostatic tachycardia syndrome), cognitive impairment and other medical conditions. Such a standardised ‘bundle’ of tests, reflecting the LC clinical characteristics and underlying mechanisms, would support not only patients in their journey, but also healthcare providers in delivering appropriate care. Structured tools such as the COVID‐19 Yorkshire Rehabilitation Scale can support symptom monitoring and guide referrals [19, 25].

### 7.2 Treatable Traits Approach

Prioritise early management of modifiable conditions such as autonomic dysfunction, which is highly disabling yet treatable. First‐line strategies include non‐pharmacological interventions (hydration, compression garments) and, where appropriate, medication [11, 25].

### 7.3 Integrated Multidisciplinary Care

Strengthen links between LC clinics, primary care and rehabilitation services to avoid fragmented pathways. Incorporate neurocognitive rehabilitation (speech and occupational therapy) alongside physical and psychological support. Evidence from the LOCOMOTION quality improvement collaborative demonstrates that streamlined referral criteria and shared learning can reduce waiting times and improve equity of access [19]. Ensure that the holistic and other advantages of MDT working do not exclude patients from shared decision making and so disempower them. ‘Nothing about me without me’ can be protected by making space for patients in MDT meetings; or at least by ensuring that key decisions are only made in meetings at which patients are present.

### 7.4 Patient‐Centred and Accessible Services

Offer hybrid models (face‐to‐face and telehealth) to improve access, particularly for rural and underserved populations. Enable patient‐initiated follow‐up and provide clear re‐entry pathways when new treatments or complications arise [2, 6].

### 7.5 Preventive Strategies

Advise on reinfection prevention (e.g., ventilation, masking). Monitor for cardiovascular, metabolic, inflammatory and other systemic complications associated with LC [30, 31, 32]. Also be alert to the exacerbation of existing health problems as early intervention can inhibit these [32].

### 7.6 Periodic Review

Keep under review those LC patients who have not recovered, instead of discharging them. Previous research suggests that LC services will need to support the long‐term impacts and complexities associated with ‘long’ (or persistent) LC [28].

Based on patients’ priorities and emerging evidence, implications for policy makers on future planning for LC clinics include:

### 7.7 Avoiding Postcode Lottery

A major take‐home from the people we spoke to about their LC clinic experiences was the importance of geographical consistency. This adds to previous research highlighting the variability in LC services across the United Kingdom, with no clearly identified best practice model [13, 27, 33]. Recent studies advocate that gold standard of care should include a multidisciplinary approach to the assessment and management of LC [10]. Furthermore, the standardisation of care facilitates monitoring of clinics’ outcome data and makes comparisons of clinics possible.

### 7.8 Financial Sustainability of LC Services

Maintain LC care in the face of NHS funding constraints and clinic closures. Core elements that should be preserved include: multidisciplinary assessment and rehabilitation, as recommended by WHO and NICE, ensuring access to physicians with relevant expertise alongside allied health professionals; [1] continuity of care to prevent patients from being lost in fragmented systems; vocational rehabilitation and psychosocial support, critical for recovery and return to work; [33] integration with primary care, enabling early screening and management of treatable traits such as autonomic dysfunction even when specialist clinics are reduced [19]. The Scottish Government's commitment of £4.5 million recurring funding for LC, ME/CFS, and similar conditions provides a model for sustainability through multi‐board collaboration and shared resources [33]. This approach—combining specialist hubs with community‐based rehabilitation and telehealth—can maintain equity and resilience even under financial constraints [34, 35].

### 7.9 Education and Evidence Uptake

Equip clinicians with up‐to‐date knowledge on LC pathophysiology and emerging treatments. Owen and Faghy emphasise the systemic barriers patients face in accessing NHS care, underscoring the need for clinician education and service redesign to reduce delays and improve diagnostic confidence [10, 36]. There was concern in our sample about the slow uptake of LC research into clinical practice. This links to the rehabilitation‐heavy approach of many LC clinics, whilst up to date evidence suggests that medical investigations and treatments also have a prime role to play. Clinical guidelines need to be updated (NICE guidelines, for instance, have had no substantive update since 2021), so that clinicians are confident to treat what is treatable [11, 25, 32]. A service that is seen to be up to date with the research evidence is more likely to inspire confidence in people using LC services and provide ‘psychological safety’, which is paramount for physical health and wellbeing [37, 38].

### 7.10 Integrated Care Beyond LC

Our study also informs the development of integrated care models for other multisystem long‐term conditions. LC shares some features with ME/CFS and post‐viral syndromes, including fluctuating symptoms, multi‐organ involvement, and the need for coordinated, multidisciplinary management. The Scottish policy to expand LC services to include ME/CFS and related conditions underscores the relevance of our recommendations beyond LC [39]. Principles such as patient‐led governance, proactive screening for treatable traits, and hybrid delivery models (face‐to‐face and digital) can guide sustainable frameworks for complex chronic conditions. These models align with international calls for integrated, person‐centred care and could serve as exemplars for managing multimorbidity in resource‐limited health systems, [1, 19, 36] particularly for those from socially excluded populations [40].

## 8 National and International Relevance

Although our findings are rooted in the UK context, they reflect global challenges in LC care. A survey of 1015 healthcare professionals across 110 countries revealed substantial variation in management approaches, shaped by resource availability, geography, and income level, with differences in multidisciplinary team (MDT) use and telemedicine adoption [41]. These disparities highlight the importance of a standardised set of initial investigations, as recommended by Nigro to rule out serious complications and identify treatable conditions early [41]. Evidence from Germany shows high variability in LC outpatient clinics and calls for unified diagnostic and therapeutic protocols, [42] while US and Australian studies emphasise telehealth and hybrid models to improve access in rural and underserved settings [2, 12]. Our proposed model—grounded in WHO guidance for assessment and rehabilitation—offers a transferable framework that prioritises equity, diagnostic clarity, and integration of emerging evidence. Similar to chronic disease models such as diabetes, this approach can be adapted across diverse healthcare systems to reduce variability and improve outcomes globally [1, 12, 19].

## 9 Future Research Priorities

This study has highlighted the need for further research in four main areas. First, there is an urgent need to revisit the NICE guidelines [9]. These were last updated in November 2021, at a time when LC had been recognised as a condition for less than 18 months. Since then, the evidence base around how to care for people with LC has increased enormously. Second, the character of LC has changed since the LOCOMOTION study: it is now predominantly a chronic condition (‘long’ LC) and so there is a pressing need for translational and clinical research into the health needs of people who have had it for more than a year. Third, the health service environment has changed greatly in the last 5 years—in the United Kingdom and elsewhere—and this creates a need for fresh service evaluations of LC care in this new context. And finally, we need a programme of equity research that understands and targets inequalities in LC: inequalities in the epidemiology, clinical manifestations, service access and health outcomes [13, 27, 33, 43, 44, 45, 46].

## 10 Conclusion

This study, based on what service users, providers and researchers think LC care should look like, fills a gap in the evidence of providing standardised principles of care for LC. These principles cover healthcare access, investigations (including red flags), screening and treatment of treatable traits, management of symptoms, providing up‐to‐date evidence‐based care, and how clinic systems function. Future researchers are encouraged to empirically test how developing standardised principles of care for LC could lead to more satisfactory patient outcomes, value for money for healthcare funders and potentially also savings to public finances.

**The LOCOMOTION Consortium**

**Table:** 

First name	Last name	Role
Nawar	Bakerly	Principal Investigator
Kumaran	Balasundaram	NHS Clinical Research Fellow
Megan	Ball	NHS Clinical Research Fellow
Mauricio	Barahona	Co‐Investigator
Alexander	Casson	Co‐Investigator
Jonathan	Clarke	HEI Researcher
Karen	Cook	Patient Advisory Group Member
Rowena	Cooper	NHS Clinical Research Fellow
Vasa	Curcin	Co‐Investigator
Julie	Darbyshire	Co‐Investigator
Helen	Davies	Principal Investigator
Helen	Dawes	Co‐Investigator
Simon	de Lusignan	Co‐Investigator
Brendan	Delaney	Chief Investigator
Carlos	Echevarria	Principal Investigator
Sarah	Elkin	Principal Investigator
Ana Belen	Espinosa Gonzalez	HEI Researcher
Rachael	Evans	Principal Investigator
Sophie	Evans	Patient Advisory Group Member
Zacchaeus	Falope	Principal Investigator
Ben	Glampson	HEI Researcher
Madeline	Goodwin	Research Assistant
Trish	Greenhalgh	Co‐Investigator
Darren C	Greenwood	Co‐Investigator
Stephen	Halpin	Principal Investigator
Juliet	Harris	NHS Research Assistant
Will	Hinton	HEI Researcher
Mike	Horton	Co‐Investigator
Samantha	Jones	NHS Clinical Research Fellow
Joseph	Kwon	HEI Researcher
Cassie	Lee	NHS Clinical Research Fellow
Ashliegh	Lovett	NHS Clinical Research Fellow
Mae	Mansoubi	HEI Researcher
Victoria	Masey	NHS Clinical Research Fellow
Harsha	Master	Principal Investigator
Erik	Mayer	HEI Researcher
Bernardo	Meza‐Torres	HEI Researcher
Ruairidh	Milne	Patient Advisory Group Member
Ghazala	Mir	Co‐Investigator
Jacqui	Morris	Principal Investigator
Adam	Mosley	NHS Research Assistant
Jordan	Mullard	HEI Researcher
Daryl	O'Connor	Co‐Investigator
Rory	O'Connor	Co‐Investigator
Thomas	Osborne	Project Manager
Amy	Parkin	NHS Clinical Research Fellow
Stavros	Petrou	Co‐Investigator
Anton	Pick	Principal Investigator
Denys	Prociuk	HEI Researcher
Clare	Rayner	Patient Advisory Group Member
Amy	Rebane	Patient and Public Involvement Manager
Natalie	Rogers	Patient Advisory Group Member
Janet	Scott	Principal Investigator
Manoj	Sivan	Chief Investigator
Nikki	Smith	Patient Advisory Group Member
Adam	Smith	Statistician
Emma	Tucker	Principal Investigator
Ian	Tucker‐Bell	Patient Advisory Group Member
Paul	Williams	NHS Clinical Research Fellow
Darren	Winch	Patient Advisory Group Member
Conor	Wood	NHS Research Assistant

## Author Contributions

**Clare Rayner:** conceptualisation, funding acquisition, methodology, validation, writing – review and editing. **Ghazala Mir:** conceptualisation, funding acquisition, validation, writing – review and editing. **Johannes de Kock:** methodology, validation, writing – review and editing, funding acquisition, conceptualisation. **Nikki Smith:** conceptualisation, methodology, writing – review and editing, supervision, funding acquisition. **Ruairidh Milne:** conceptualisation, methodology, funding acquisition, validation, formal analysis, supervision, investigation, writing – original draft, writing – review and editing. **Nawar Diar Bakerly:** conceptualisation, methodology, funding acquisition, writing – original draft, formal analysis.

## Ethics Statement

Patient and staff participants gave written informed consent. The views expressed in this publication are those of the author(s) and not necessarily those of NIHR or The Department of Health and Social Care.

## Conflicts of Interest

The authors declare no conflicts of interest.

## Supporting information

supmat.

## Data Availability

The data that support the findings of this study are available on request from the corresponding author. The data are not publicly available due to privacy or ethical restrictions.

## References

[1] Organization WH , “Clinical Management of COVID‐19: Living Guideline,” 15 September 2022. World Health Organization; 2022, https://iris.who.int/handle/10665/362783. 35917394

[2] E. W. Ely , L. M. Brown , and H. V. Fineberg , “Long Covid Defined,” New England Journal of Medicine 391, no. 18 (2024): 1746–1753, 10.1056/NEJMsb2408466. 39083764 PMC11687645

[3] H. V. Fineberg , L. Brown , T. Worku , and I. Goldowitz , *A Long COVID Definition: A Chronic, Systemic Disease State With Profound Consequences* (National Academies Press, 2024), 10.17226/27768. 39110819

[4] H. E. Davis , L. McCorkell , J. M. Vogel , and E. J. Topol , “Long COVID: Major Findings, Mechanisms and Recommendations,” Nature Reviews Microbiology 21, no. 3 (2023): 133–146, 10.1038/s41579-022-00846-2. 36639608 PMC9839201

[5] C. E. Hastie , D. J. Lowe , A. McAuley , et al., “True Prevalence of Long‐COVID in a Nationwide, Population Cohort Study,” Nature Communications 14, no. 1 (2023): 7892, 10.1038/s41467-023-43661-w. PMC10689486 38036541

[6] “Self‐Reported Coronavirus (COVID‐19) Infections and Associated Symptoms, England and Scotland—Office for National Statistics,” accessed January 19, 2025, https://www.ons.gov.uk/peoplepopulationandcommunity/healthandsocialcare/conditionsanddiseases/datasets/selfreportedcoronaviruscovid19infectionsandassociatedsymptomsenglandandscotland.

[7] J. Kwon , R. Milne , C. Rayner , et al., “Impact of Long COVID on Productivity and Informal Caregiving,” European Journal of Health Economics 25, no. 7 (2024): 1095–1115, 10.1007/s10198-023-01653-z. PMC11377524 38146040

[8] “The‐Economic‐Burden‐of‐Long‐Covid‐in‐the‐UK_Cambridge‐Econometrics_V1.1_March2024.”

[9] Overview | COVID‐19 Rapid Guideline: Managing the Long‐Term Effects of COVID‐19 | Guidance | NICE, accessed July 29, 2023, https://www.nice.org.uk/guidance/ng188.

[10] S. Rajan , K. Khunti , N. Alwan , et al., In the Wake of the Pandemic: Preparing for Long COVID, 2021, Policy Brief (39): 1–27, accessed January 19, 2025 2021, https://www.ncbi.nlm.nih.gov/books/NBK569598/. 33877759

[11] H. Lewthwaite , A. Byrne , B. Brew , and P. G. Gibson , “Treatable Traits for Long COVID,” Respirology 28, no. 11 (2023): 1005–1022, 10.1111/resp.14596. 37715729

[12] NHS England, Commissioning Guidance for Post‐COVID Services for Adults, Children and Young People, accessed January 19, 2025, https://www.england.nhs.uk/long-read/commissioning-guidance-for-post-covid-services-for-adults-children-and-young-people/.

[13] T. Greenhalgh , J. L. Darbyshire , C. Lee , E. Ladds , and J. Ceolta‐Smith , “What Is Quality in Long Covid Care? Lessons From a National Quality Improvement Collaborative and Multi‐Site Ethnography,” BMC Medicine 22, no. 1 (2024): 159, 10.1186/s12916-024-03371-6. 38616276 PMC11017565

[14] J. Mullard , G. Mir , C. Herbert , and S. Evans , “You're Just a Guinea Pig’: Exploring the Barriers and Impacts of Living With Long COVID‐19: A View From the Undiagnosed,” Sociology of Health & Illness 46, no. 8 (2024): 1602–1625, 10.1111/1467-9566.13795. 38850204

[15] E. Duan , K. Garry , L. I. Horwitz , and H. Weerahandi , “I Am Not the Same as I Was Before”: A Qualitative Analysis of COVID‐19 Survivors,” International Journal of Behavioral Medicine 30, no. 5 (2023): 663–672, 10.1007/s12529-022-10129-y. 36227557 PMC9559269

[16] LOCOMOTION—Long COVID Study, https://locomotion.leeds.ac.uk/.

[17] M. Sivan , T. Greenhalgh , J. L. Darbyshire , et al., “LOng COvid Multidisciplinary Consortium Optimising Treatments and Services Across the NHS (LOCOMOTION): Protocol for a Mixed‐Methods Study in the UK,” BMJ Open 12, no. 5 (2022): e063505, 10.1136/BMJOPEN-2022-063505. PMC9114312 35580970

[18] A. Tong , P. Sainsbury , and J. Craig , “Consolidated Criteria for Reporting Qualitative Research (COREQ): A 32‐Item Checklist for Interviews and Focus Groups,” International Journal for Quality in Health Care 19, no. 6 (2007): 349–357, 10.1093/intqhc/mzm042. 17872937

[19] J. Darbyshire , T. Greenhalgh , N. D. Bakerly , et al., “Improving Quality in Adult Long Covid Services: Findings From the LOCOMOTION Quality Improvement Collaborative,” Clinical Medicine 24, no. 5 (2024): 100237, 10.1016/j.clinme.2024.100237. 39181334 PMC11421994

[20] Health Organization W . “Guideline Clinical Management of COVID‐19 Patients: Living Guideline,” 18 November 2021. Published Online 2021.

[21] Safe Rehabilitation Approaches for People Living With Long Covid: Physical Activity and Exercise Acknowledgement. Published Online 2021.

[22] V. Braun and V. Clarke , “Using Thematic Analysis in Psychology,” Qualitative Research in Psychology 3, no. 2 (2006): 77–101, 10.1191/1478088706qp063oa.

[23] T. Greenhalgh , M. Sivan , A. Perlowski , and J. Nikolich , “Long COVID: A Clinical Update,” Lancet 404, no. 10453 (2024): 707–724, 10.1016/S0140-6736(24)01136-X. 39096925

[24] “What Can We Learn About Long Covid Care From Health Records Data,” accessed February 2, 2025, https://locomotion.leeds.ac.uk/news/locomotion-webinar-health-records-data/.

[25] N. Diar Bakerly , N. Smith , J. L. Darbyshire , et al., “Pathophysiological Mechanisms in Long COVID: A Mixed Method Systematic Review,” International Journal of Environmental Research and Public Health 21, no. 4 (2024): 473, 10.3390/ijerph21040473. 38673384 PMC11050596

[26] S. A. Baz , C. Fang , J. D. Carpentieri , and L. Sheard , “‘I Don't Know What to Do or Where to Go’. Experiences of Accessing Healthcare Support From the Perspectives of People Living With Long Covid and Healthcare Professionals: A Qualitative Study in Bradford, UK,” Health Expectations 26, no. 1 (2023): 542–554, 10.1111/hex.13687. 36512382 PMC10124541

[27] C. Fang , S. A. Baz , L. Sheard , and J. D. Carpentieri , ““They Seemed to Be Like Cogs Working in Different Directions”: A Longitudinal Qualitative Study on Long COVID Healthcare Services in the United Kingdom From a Person‐Centred Lens,” BMC Health Services Research 24, no. 1 (2024): 406, 10.1186/s12913-024-10891-7. 38561719 PMC10986002

[28] D. Sunkersing , M. Ramasawmy , N. A. Alwan , et al., “What Is Current Care for People With Long COVID in England? A Qualitative Interview Study,” BMJ Open 14, no. 5 (2024): e080967, 10.1136/bmjopen-2023-080967. PMC11107429 38760030

[29] F. Turk , J. Sweetman , C. A. Chew‐Graham , M. Gabbay , J. Shepherd , and C. van der Feltz‐Cornelis , “Accessing Care for Long Covid From the Perspectives of Patients and Healthcare Practitioners: A Qualitative Study,” Health Expectations 27, no. 2 (2024): e14008, 10.1111/hex.14008. 38481384 PMC10938067

[30] Y. W. Heo , J. J. Jeon , M. C. Ha , Y. H. Kim , and S. Lee , “Long‐Term Risk of Autoimmune and Autoinflammatory Connective Tissue Disorders Following COVID‐19,” JAMA Dermatology 160, no. 12 (2024): 1278, 10.1001/jamadermatol.2024.4233. 39504045 PMC11541739

[31] S. H. Lim , H. J. Ju , J. H. Han , et al., “Autoimmune and Autoinflammatory Connective Tissue Disorders Following COVID‐19,” JAMA Network Open 6, no. 10 (2023): e2336120, 10.1001/jamanetworkopen.2023.36120. 37801317 PMC10559181

[32] Z. Al‐Aly , Y. Xie , and B. Bowe , “High‐Dimensional Characterization of Post‐Acute Sequelae of COVID‐19,” Nature 594, no. 7862 (2021): 259–264, 10.1038/s41586-021-03553-9. 33887749

[33] E. Duncan , L. Alexander , J. Cowie , et al., “Investigating Scottish Long COVID Community Rehabilitation Service Models From the Perspectives of People Living With Long COVID and Healthcare Professionals: A Qualitative Descriptive Study,” BMJ Open 13, no. 12 (2023): e078740, 10.1136/bmjopen-2023-078740. PMC10729197 38101833

[34] S. H. Bardach , J. D. Lichtenstein , F. Velcani , et al., “A Post‐Acute COVID‐19 Syndrome (PACS) Clinic in Rural New England,” American Journal of Medical Quality 39, no. 5 (2024): 244–250, 10.1097/JMQ.0000000000000202. 39268907

[35] L. Cummings , “Long COVID: The Impact on Language and Cognition,” Language and Health 1, no. 1 (2023): 2–9, 10.1016/j.laheal.2023.05.001.

[36] S. Luo , Z. Zheng , S. R. Bird , et al., “An Overview of Long COVID Support Services in Australia and International Clinical Guidelines, With a Proposed Care Model in a Global Context,” Public Health Reviews 44 (2023): 1606084, 10.3389/phrs.2023.1606084. 37811128 PMC10556237

[37] L. Morton , N. Cogan , J. Kolacz , et al., “A New Measure of Feeling Safe: Developing Psychometric Properties of the Neuroception of Psychological Safety Scale (NPSS),” Psychological Trauma: Theory, Research, Practice, and Policy 16, no. 4 (2024): 701–708, 10.1037/tra0001313. 35849369

[38] J. Spinazzola , B. van der Kolk , and J. D. Ford , “When Nowhere Is Safe: Interpersonal Trauma and Attachment Adversity as Antecedents of Posttraumatic Stress Disorder and Developmental Trauma Disorder,” Journal of Traumatic Stress 31, no. 5 (2018): 631–642, 10.1002/jts.22320. 30338544 PMC6221128

[39] Funding Long COVID Services ‐ gov.scot, accessed December 7, 2025, https://www.gov.scot/news/funding-long-covid-services/.

[40] G. Mir , J. Mullard , A. Parkin , et al., “Addressing Inequalities in Long Covid Healthcare: A Mixed‐Methods Study on Building Inclusive Services,” Health Expectations 28, no. 4 (2025): e70336, 10.1111/hex.70336. 40600494 PMC12215820

[41] M. Nigro , C. Valenzuela , F. Arancibia , et al., “A Worldwide Look Into Long COVID‐19 Management: An End‐COVID Survey,” ERJ Open Research 10, no. 6 (2024): 00096‐2024, 10.1183/23120541.00096-2024. 39534773 PMC11551856

[42] L. C. Adam , F. Boesl , V. Raeder , et al., “The Legacy of the COVID‐19 Pandemic for the Healthcare Environment: The Establishment of Long COVID/Post‐COVID‐19 Condition Follow‐Up Outpatient Clinics in Germany,” BMC Health Services Research 25, no. 1 (2025): 360, 10.1186/s12913-025-12521-2. 40065313 PMC11895281

[43] H. E. Davis , G. S. Assaf , L. McCorkell , et al., “Characterizing Long COVID in an International Cohort: 7 Months of Symptoms and Their Impact,” EClinicalMedicine 38 (2021): 101019, 10.1016/j.eclinm.2021.101019. 34308300 PMC8280690

[44] C. Tackey , P. M. Slepian , H. Clarke , and N. Mittal , “Post‐Viral Pain, Fatigue, and Sleep Disturbance Syndromes: Current Knowledge and Future Directions,” Canadian Journal of Pain 7, no. 2 (2023): 2272999, 10.1080/24740527.2023.2272999. 38239826 PMC10795785

[45] J. Choutka , V. Jansari , M. Hornig , and A. Iwasaki , “Unexplained Post‐Acute Infection Syndromes,” Nature Medicine 28, no. 5 (2022): 911–923, 10.1038/s41591-022-01810-6. 35585196

[46] C. M. van der Feltz‐Cornelis , J. Sweetman , F. Turk , et al., “Integrated Care Policy Recommendations for Complex Multisystem Long Term Conditions and Long COVID,” Scientific Reports 14, no. 1 (2024): 13634, 10.1038/s41598-024-64060-1. 38871773 PMC11176166

