# Supplementary material for: ‘What Do People With Long Covid Want From Healthcare Services?’ A Qualitative Exploration From Lived Experience
Source: Health Expect. 2026 Feb 24;29(2):e70607. doi: 10.1111/hex.70607 (PMC12932910; doi:10.1111/hex.70607)
Supplement: Supplementary file 1 — supmat. [file HEX-29-e70607-s001.docx]

**Online appendix (1). Full list of participants’ quotes**

*(1) Who the services are for - access to healthcare*

a. People with LC, however long they have had it – not just those newly identified.

“It is becoming clear that a sizeable proportion of people with LC therefore have a chronic condition that needs long term management, and the needs of these patients are different from, say, those who get better within 9 months. We need a working definition for ‘Long LC’. In terms of pathology, a starter would be anyone still unwell more than 12 months after infection”. The duration becomes essential when considering the economic burden in the UK of long term work absence in the prime working age groups. [PAG member]

b. A focus on reducing inequalities and stigma.

Patient participants expressed a need for a straightforward process to access clinics.

“The referral process is complicated, it gets lost, or rejected. You can only get through if you ring up each day or really make a nuisance of yourself.” [PAN member]

“Patients often have to coordinate their own care.” [PAN member]

“It’s easier if you are well educated.” [PAN member]

“…some doctors not knowing that the clinics exist or not knowing how to refer a patient to them.” [PAN member]

“It was very hard beginning to get doctors to understand what we were going through. When I did get results of a scan showing blood clot and heart information I got a lot more support because the problems were visible. I think the hardest thing is for patients who don’t have those results to get the support they need. It’s really important to find good pathways for patients.” [PAG member]

“My worst experience was lack of any clinical input by health services when I was very ill with acute Covid. I am ‘CEV’ (Clinically Extremely Vulnerable - [a designation by UK government in early 2020 that denoted someone with underlying illness which made them particularly vulnerable to developing severe complications from COVID). My specialists refused to see me because I was not admitted to hospital, they said there couldn’t be anything wrong with me if I hadn’t been admitted. I should have been, I just didn’t have respiratory problems”. [PAG member]

“I had a red mark against my name at the GP’s, with written instruction “You are not allowed to contact us about this issue again”.” [PAN member]

“LC is not an easy name. LC as a term closes people's minds. Some doctors said “don’t mention the word LC-it's just anxiety”.” [PAN member]

“Best to explain what symptoms you have that have started since infection, and these can be investigated.” [PAN member]

*(2) What services do.*

a. Systems

[Those providing healthcare services for people with LC] “need to understand best practice for treating multiple symptoms together. A multi-condition approach is key. This may be best branded as ‘integrated multi system rehabilitation’” [Consultant Physician].

b. Consistency in investigations and treatments

“[We need] consistency in testing and diagnosis. Currently there are a variety of healthcare professionals involved in testing and diagnosis – no real consistency, it’s more based upon an individual clinician’s knowledge”. [EAG member]

“Above all, people need the right tests at the right time and to consider the known range of COVID-19-related problems.” [PAG member]

“Other illnesses should be ruled out.” [PAG member]

Patients also want “mental health support because of the effect on finances and work and the difficulties of living with a long-term disease” and “signposting to relevant support e.g. via social prescribing systems.” [PAG member]

“Whilst there may be specific community needs that might necessitate minor adjustments to what a LC clinic offers, there are many aspects of the condition that remain relevant across the board and this should be reflected in the services provided nationwide.” [PAG member]

“I was advised to do a graded increase in my exercise which caused me to crash and ended up back in hospital. We should only be told to do a low level of exertion initially. Starting with respiratory muscle retraining or breathing exercises.” [PAG member]

There was a range of attitudes towards the number of tests that patients would like. Some wanted a detailed list of tests which reflected current research findings. “The acceptability of tests is fairly high, as people are desperate and will go through the motions in order to rule things out and get to a diagnosis.” [EAG member]

Others wanted only those that are “likely to have a yield” [PAN member]

“I don’t want a lot of tests - just ones which may be useful.” [PAN member]“

“Investigate and treat chest pain.” [PAG member]

“…appropriate tests relevant to Long COVID - can GPs start these off?” [PAN member]

“Please do rule out significant conditions that can occur with COVID. Remember it’s a diagnosis of exclusion you’ve got to do those tests and rule things out, like large blood clots.” [PAG member]

“It is critical that we understand the pathology and find treatments. Meantime, we want treatable conditions to be identified and treated. We would like clinicians to keep up with the research and put this into clinical practice. For people who are not better to be reassessed at intervals, and keep an open mind.” [PAG member]

c. Trying treatments.

“The end goal is to better understand how to treat people.” [PAG member]

“I would like clinicians to find the gold standard of care.” [PAG member]

“The most important thing is that research is translated into clinical practice as soon as possible.” [PAG member]

“We ask please hear us, involve us, but also be brave, try things that may not necessarily fit onto an NHS tick list.” [PAG member]

**Autonomic Dysfunction (AD)**

“Focus on recommendations that would be easy for clinics to implement and have a quick impact on patients: an easy win”. [(Co-Chief Investigator])

“Postural tachycardia syndrome and low blood pressure are common. It’s symptomatic, highly disabling and treatable.” The diagnosis of AD is seen as a passport to self-management, treatment and credibility.” [PAN member]

“Once diagnosis and support are offered, POTS becomes easier (to an extent) to live with, as you can manage the symptoms and know when an attack is coming .” [PAN member]

“Diagnosis is important because without it I would hardly be able to get out of bed because of how faint I felt.” [PAN member]

“Without a diagnosis of autonomic dysfunction, you are unable to access meds.” [PAN member]

“The improvement in my symptoms with treatment is huge: life-changing.” [PAN member]

“Treatment has helped blood pressure and fatigue.” [PAN member]

“Having a named diagnosis (e.g. POTS) is important to patients - it needs to be acknowledged this is also down to being able to share something with healthcare professionals which they will understand.” [PAN member]

“Being able to give healthcare professionals a named condition helps them with how they provide treatment and support, especially those who view LC as psychosomatic and/or don’t know anything about it.” [PAN member]

“It is okay to ask LC patients to monitor their blood pressure at home as part of POTS testing/diagnosis. They may need signposting for where they can get a monitor from if they are unable to provide their own e.g. borrow from GP Practice.” “It’s easier for patients to test themselves for POTS by home monitoring of blood pressure and record their results.” [PAN member]

**Cognitive issues**

“I think there is not enough being done especially in the world of cognitive and neurological issues. It was always labelled as brain fog. Now having lived with it for 2.5 years I see a difference between neurological and cognitive issues and brain fog.” [PAG member]

“My cognitive problems were not dealt with because I was told I did not have dementia.” [PAG member]

“When I said that my memory was badly affected the neurologist responded by saying “don’t worry about that” with a flick of his hand as if to brush it away.” [PAG member]

“Speech therapy for Covid-related speech problems needs a neuro-cognitive approach.” [PAG member]

d. Patient review

“What tests need repeating and when? e.g. cardiac, neuro, respiratory, GI, autonomic?” [PAG member]

“How do we ensure that COVID as a cardiac risk factor is recorded?” [PAG member]

“Are people post discharge getting better, staying the same or getting worse?” [PAG member]

*(3) How services operate.*

a. Coordination of care

“The integration and collaboration of community and specialist services allowed us to share feedback more easily and refer patients more quickly.” [Consultant Physician]

“We would like to provide more LC support in-house, as it saves referral time.” [Consultant Physician]

“…a one-stop shop - having everything in the same place, it’s simple to touch base with all the treatment they are receiving.” [PAN member]

“I want to be informed where I am on the referral pathway, so I don’t have to chase to find out - bearing in mind our cognitive dysfunction!” [PAG member]

“Medical Silos are a problem - being left high and dry if a specialist discharges, instead of back to the LC service which has a good understanding of all our problems.” [PAG member]

“When I was discharged from the Long COVID clinic I felt abandoned and adrift. This illness had caused me to lose my job. It was affecting my life in so many different ways.” [PAG member]

“Clinics need a mechanism for getting in touch with LC patients when new treatments and tests become available. Or regular review, e.g. every year or two years - to look for new problems or problems that might have been missed previously.” [PAN member]

‘Patient-initiated follow-up by the LC clinic can work. Or a coordinating clinic (such as in Paediatric services) who have a ‘holding pattern’ i.e. before discharge a patient could first touch base with the LC service. [Consultant Physician]

“Clinics need a mechanism for getting in touch with LC patients when new treatments and tests become available. Or regular review, e.g. every year or two years - to look for new problems or problems that might have been missed previously.” [PAN member]

b. Proactive

“Patients shouldn’t have to push for what there is already evidence of e.g. the right tests.” [PAN member]

“Most clinics are about rehab, not investigation or treatment – frustration with that. (Though specific aspects of rehab can be very helpful).” [PAN member]

“There is certain advice that everyone can be given whilst waiting to be seen - early pacing advice is one of the most helpful things”. [PAN member]

“It would be good to be given a sheet about PoTS symptoms whilst you’re waiting for tests. It’s a good opportunity to monitor your own symptoms and try things that may help. Peer support groups can be really effective for this.” [PAN member]

“Reinfections are damaging and need to be prevented to reduce the overall impact of the illness on daily functions”. [PAG member]

“We want to avoid reinfections - how to avoid (masks, ventilation, work adjustments etc), how to manage if they happen (metformin, enough rest etc.).” [EAG member]

“We also want prevention of cardiovascular complications that are associated with LC.” [PAG member]

“Digital technology could be used even more. “It would be helpful for GPs to access”. [PAN member]

“Newer clinics benefit from sharing best practice and existing resources so they don’t have to design them from scratch themselves – collaboration is key”. [Data specialist]

c. ‘Evidence-based’, but creatively not rigidly: thoughtful, agile, fleet of foot

“It is a recurring theme in patient groups that healthcare for LC lags behind the research evidence.” [PAG member]

“Education is needed so that LC service staff are up to date on research: education influences just about everything – assessments, investigations/tests, advice, treatments, monitoring.” [PAG member]

“We want clinicians to base their diagnostic and therapeutic decisions on best available evidence, and there is now lots, in discussion with patients. Not to be paralysed by out of date evidence (NICE, 2021) or the lack of RCTs. We need clinicians to be agile and fleet-of-foot.” [PAG member]

“As clinics close, there is real concern that the expertise gained over the last few years is going to be lost.” [PAG member]

d. Genuinely patient centred and listening to patients, even multi-disciplinary team meetings (MDTs)MDTs

“My clinic was a really good experience with a holistic approach with tests, referrals, health education and teaching of self-management techniques.” [PAN member]

“There is an obvious physical impact but there is the emotional impact as well.” [PAN member]

“It helped when there was acknowledgement by staff of the effects of LC”. [PAN member]

“Our experience is much better when the patient is put at the centre of diagnosis and support and has things properly explained to them. Even though the ultimate outcome of living with a long-term health condition is the same, the delivery of support can empower the patient to better manage their condition.” [PAN member]

“I think you really need to listen to your patient if they say they are completely different from before they got ill, they need to be listened to. It should not be passed over as anxiety or needing exercise, you really need to listen to what they’re struggling with and try to refer them to the appropriate team.” [PAG member]

“Learning from colleagues, upskilling within teams, improved continuity of care and a better awareness of the full range of symptoms which apply to LC patients.”. [Consultant Physician]

“The term multi-disciplinary team in my experience is often used by managers and policy makers to refer to non-doctors i.e. nurses, physios etc. It’s confusing because doctors see themselves as part of that team. We therefore need to be careful about the term and be careful how we use it ourselves”. [PAG member]
